# Expression of human cytomegalovirus components in the brain tissues of patients with Rasmussen’s encephalitis

**DOI:** 10.1007/s12250-016-3917-z

**Published:** 2017-03-02

**Authors:** Yao Zhang, Yisong Wang, Sichang Chen, Shuai Chen, Yuguang Guan, Changqing Liu, Tianfu Li, Guoming Luan, Jing An

**Affiliations:** 1 Department of Neurosurgery, Sanbo Brain Hospital, Capital Medical University, Beijing, 100093 China; 2 Department of Microbiology, School of Basic Medical Science, Capital Medical University, Beijing, 100069 China; 3 Beijing Key Laboratory of Epilepsy, Beijing, 100093 China; 4 Center of Epilepsy, Beijing Institute for Brain Disorders, Beijing, 100093 China

**Keywords:** Rasmussen’s encephalitis, human cytomegalovirus (HCMV), pp65, epilepsy

## Abstract

Rasmussen’s encephalitis (RE) is a rare and severe progressive epileptic syndrome with unknown etiology. Infection by viruses, including human cytomegalovirus (HCMV), has been speculated to be a potential trigger for RE. However, no viral antigens have been detected in the brains of patients with RE; thus, a possible clinical linkage between viral infections and RE has not been firmly established. In this study, we evaluated the expression of HCMV pp65 antigen in brain sections from 26 patients with RE and 20 non-RE patients by immunohistochemistry and *in situ* hybridization, and assessed the associations between HCMV infection and clinical parameters. Elevated expression of HCMV pp65 protein and DNA was observed in 88.5% (23/26) and 69.2% (18/26) of RE cases, respectively. In the non-RE group, HCMV pp65 antigen was detected only in two cases (10%), both of which were negative for DNA staining. Additionally, the intensity of HCMV pp65 staining was correlated with a shorter duration of the prodromal stage, younger age of seizure onset, and more severe unilateral cortical atrophy. Elevated expression of HCMV pp65 was observed in RE brain tissue and was correlated with the clinical features of RE disease. In summary, our results suggested that HCMV infection may be involved in the occurrence and progression of RE disease. Thus, further studies are needed to determine whether early treatment with anti-HCMV antibodies could modulate the course of RE.

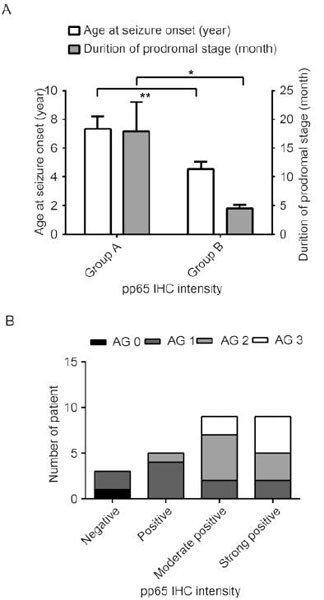

## INTRODUCTION

Rasmussen's encephalitis (RE) is a rare, chronic, progressive neurological syndrome that often occurs in childhood (Varadkar et al., 2014). The major clinical characteristics of RE consist of focal epilepsy, progressive hemiparesis, and the decline of cognitive function (Bien et al., 2002b). Without surgical intervention, patients with RE eventually develop to a persistent status of epilepsy, with more severe deterioration of neurological function and cognitive impairment (Bien et al., 2002b). Currently, hemispherectomy is the only effective treatment for controlling seizures and cognitive deterioration (Guan et al., 2014).

The etiology and pathogenesis of RE are unclear (Varadkar et al., 2014). Several factors are thought to be involved in the occurrence of this syndrome, including virus infection (Walter and Renella, 1989; Farrell et al., 1991), autoantibodies (Rogers et al., 1994), cytotoxic T cells (Bauer et al., 2002; Bien et al., 2002a), and abnormal immune modulation (Luan et al., 2013; Luan et al., 2016). Since various pathological characteristics observed in the brains of patients with RE, such as lymphocyte infiltration, neuron loss, vascular cuffing, and microgliosis (Pardo et al., 2004), are similar to those observed in viral encephalitis, virus infection has been proposed to be an important cause of RE (Rasmussen et al., 1958). This hypothesis was supported by data from previous studies in which nucleic acids of human cytomegalovirus (HCMV), herpes simplex virus 1(HSV-1), and Epstein-Barr virus (EBV) were detected in the brain tissues of patients with RE by *in situ* hybridization or polymerase chain reaction (PCR), although viral antigen has not been reliably detected (Walter and Renella, 1989; Power et al., 1990; Jay et al., 1995). Moreover, Takahashi *et al*. found that nearly 50% of patients with RE had a prior history of infection or vaccination (Takahashi et al., 2006), further supporting a role of virus infection in RE. However, some researchers have failed to detect viral components (Atkins et al., 1995). Therefore, additional, more comprehensive investigations are needed to identify the presence of viral antigens and nucleic acids in the brain tissues of patients with RE, and the clinical relevance of these findings needs to be carefully analyzed.

HCMV is a double-stranded DNA virus that belongs to the family *Herpesviridae*. This virus contains approximately 200 protein-coding genes, including *pp65* and immediate-early (*IE*) genes (Tandon and Mocarski, 2012). HCMV *pp65* is the most abundant virion matrix protein and is often used as an indicator of HCMV infection. HCMV was found to have a 70%–90% prevalence in the general population and persists for the lifetime of the host after primary infection (Britt, 2015). Approximately 30% of pregnant women experiencing primary HCMV infection will transmit the virus to their developing fetuses during some stage of pregnancy, resulting in a congenital HCMV infection rate of 1%–7% at birth (Manicklal et al., 2013; Britt, 2015). Congenital HCMV infection may either be asymptomatic or cause hearing impairment, mental retardation, cerebral palsy, and neuron developmental disabilities (Manicklal et al., 2013). Whether the childhood neurological disorder of RE patients has any link to HCMV infection is thus of considerable sci-entific and clinical interest.

In the present study, we examined the expression of HCMV pp65 protein and DNA in the brain tissues of 26 patients with RE and 20 control patients without RE. We then analyzed the relationships between HCMV pp65 expression levels and clinical parameters. Our results will provide important insights for further clarifying the role of HCMV in the occurrence and progression of RE and regarding the feasibility of anti-HCMV treatment in patients with RE.

## MATERIALS AND METHODS

### Patients, diagnosis, and sample collection

Twenty-six patients with RE, who were admitted to Sanbo Brain Hospital from April 2008 to December 2013, were included in the study. The clinical diagnosis was made according to the European diagnostic criteria (Bien et al., 2005). The control group consisted of 20 patients who underwent surgical treatment for temporal lobe epilepsy, generally matched with the RE patient group for age at the time of surgery and seizure onset. After craniotomy, at least two blocks of brain tissue from different areas were collected from patients with RE. The tissues were fixed, embedded in paraffin, sliced to 6-μm thickness, and then used for pathohistological analysis and HCMV detection. In the control group, brain samples were obtained from the edges of the resected epileptogenic cortex.

### Immunohistochemical staining (IHC)

After routine preparation, the tissue sections were heated to 60 °C for antigen retrieval. Endogenous peroxidase activity and nonspecific antibody binding sites were blocked with 3% hydrogen peroxide and 1% bovine serum albumin (BSA) in phosphate-buffered saline (PBS) with 0.05% Tween 20. Subsequently, the sections were incubated with mouse anti-pp65 antibodies (ab49214, 1:200; Abcam, Cambridge, UK) or mouse anti-IE1 antibodies (ab65104, 1:40; Abcam) at 4 °C overnight. After washing, the sections were further incubated with secondary antibodies (horseradish peroxidase [HRP]-conjugated goat anti-mouse IgG). Finally, diaminobenzidine (DAB) substrate was added for coloration. The sections were then counterstained with hematoxylin and photographed (Olympus X-cite 120; Olympus, Japan).

### *In situ* hybridization (ISH)

For the detection of HCMV pp65 DNA in brain sections, a digoxin-labeled DNA probe specific for pp65 (5′-GCTCTTCTTTTTCGATATCGACTTGTTGCTGCAGCG-3′), digoxin-labeled Alu DNA probe (positive control), and insect genomic DNA probe (negative control) were purchased from Life Technologies (Eugene, USA). For the ISH procedure, after pretreatment with proteinase K (100 μg/mL) for 25 min at 37 °C, tissue sections were hybridized with the probes diluted using DIG Easy Hyb (11603558001; Rochediagnostic, Indianapolis, USA) at 95 °C for 20 min, followed by incubation for 16 h at 37 °C in a hybridization oven (UVP HL-2000

Hybrilinker). After incubation with mouse anti-digoxin IgG, the reaction was visualized by the addition of HRP-conjugated goat anti-mouse IgG and DAB, and images were acquired.

### Scoring methodology of IHC and ISH

The IHC and ISH results were evaluated by following a previously described scoring methodology (Luan and Gao et al., 2013). Cells showing yellow or brown particles in the cytoplasm or nucleus were considered positive and were counted and analyzed using image analysis software (Image-Pro Plus 6.0; Media Cybernetics Inc., Bethesda, USA). The semiquantitative results were expressed as the percentage of positive cells combined with a subjective assessment of staining intensity, which was scored as 0 (colorless), 1 (light yellow), 2 (yellow or brown), or 3 (dark brown); the percentages of positive cells were denoted as 0 (<5%), 1(5%–25%), 2 (26%–50%), 3 (51%–75%), or 4 (>75%). The product of the two scores was used to evaluate the immunostaining results, as follows: overall scores of ≤1, 2–3, 4–5, and > 6 were defined as negative, weakly positive, moderately positive, and strongly positive, respectively.

### Statistical analysis

Statistical analysis was performed using SPSS 17.0 software. Chi square tests were performed to determine the differences between the expression of HCMV components in patients with or without RE. Student's t tests and check-board analyses were performed to determine the differences in clinical features in subgroups of patients with RE, according to the pp65 IHC intensity. Differences with $P$ values of less than 0.05 were considered statistically significant.

## RESULTS

### Clinical characteristics of patients with and without RE

Twenty-six patients with RE were enrolled in this study, including 16 girls (61.5%) and 10 boys (38.5%). Their mean ages were 5.8 years (range: 1.2–18 years) and 7.5 years (range: 2.4–18.5 years) at seizure onset and at time of surgery, respectively. History of infection and/or viral vaccination prior to the seizure onset was observed in 14/26 (53.8%) patients. Eleven patients (42.3%) underwent left-side surgery, including functional hemispherectomy (FH), anatomic hemispherectomy (AH), and hemisphere disconnection. The etiologies for the control group consisted of ganglioglioma (n = 5), hippocampal sclerosis (n = 8), and focal cortical dysplasia (n = 7). The baseline comparison between RE and control groups is shown in Table 1.

Table 1. Clinical characteristics of patients with and without RE

| | RE (n = 26) | Non-RE (n = 20) |
|---|---|---|
| Gender (female) | 10 (38.5%) | 12 (60%) |
| Preceding infection | 14 (53.8%) | 3 (6%) |
| Mean age at seizure onset (years) | 5.8 | 8.6 |
| Mean age at surgery (years) | 7.5 | 14.8 |
| Side of resection (left) | 11(42.3%) | 13 (65%) |
| Surgical method | | |
| FH | 15 (46.2%) | 0 |
| AH | 2 (15.4%) | 0 |
| Disconnection | 9 (26.9%) | 0 |
| Lobectomy | 0 | 20 (100%) |

Note: FH: functional hemispherectomy; AH: anatomic hemispherectomy

### Magnetic resonance imaging (MRI) and pathological findings in the brain tissues of patients with RE

Mild to severe progressive unilateral multifocal cortical atrophy around the sylvian fissure and diffuse subcortical hypersignal confined within the involved hemisphere in the FLAIR sequence were observed in all patients (Figure 1A, 1B). The pathological changes, including pyknosis, loss of neurons, diffuse lymphocyte infiltration, perivascular lymphocyte cuffing, and glial cell proliferation (Figure 1C, 1D), were observed in RE brain tissues. However, no obvious morphological features of viral inclusion were observed.

### Expression of HCMV components in the brain tissues of patients with and without RE

Among the 26 patients with RE, 23 (88.5%) showed positive immunostaining for the HCMV pp65 protein. Among these 23 cases, nine (34.6%) were strongly positive, nine were moderately positive, and five (19.2%) were weakly positive (Figure 2, Table 2). Similarly, HCMV pp65 DNA was detected in 69.2% (18/26) of RE samples; among them, eight (30.8%), six (23.1%), and four (15.4%) were strongly, moderately, and weakly positive, respectively (Figure 3, Table 2). HCMV pp65 protein was predominantly expressed in the cytoplasm of neuron-like cells (Figure 2). In the control group, only two cases (2/20, 10%) were weakly positive for pp65 antigen, and all cases were negative for pp65 DNA (Figure 2, Table 2; $P < 0.05$ for both). Notably, all of the 18 HCMV pp65 DNA-positive RE samples were positive for HCMV pp65 protein (Table 3). In other words, five IHC-positive patients with RE showed negative ISH staining, in-

Figure 1. MRI and pathological changes in the brains of patients with RE. Atrophy of the right hemisphere cortex and widening of the sulcus (arrowheads) and caudate nucleus (arrow) were noted before operation (A). Diffuse hypersignal in the subcortical area (arrows, B). Microglial nodule formation in the cortex (arrow, C). Lymphocyte infiltration and features of perivascular cuff (arrow, D).

dicating that combined application of the two methods may be necessary for enhancement of the detection rate.

### Association between HCMV pp65 antigen expression and the clinical parameters of patients with RE

Based on the IHC intensity of HCMV pp65 antigen, patients with RE were divided into two groups: group A with negative or weakly positive pp65 staining, and group B with moderately or strongly positive pp65 staining. As shown in Figure 4, patients in group B showed a shorter mean duration of the prodromal stage (17.9 ± 5.1 versus 4.6 ± 0.56 months, respectively; Figure 4A; $P < 0.05$) and a younger age at seizure onset as compared with group A patients (4.5 ± 0.5 versus 7.4 ± 0.9 years, respectively; Figure 4A; $P < 0.01$). Additionally, patients with RE with more intense HCMV pp65 staining showed a more severe hemisphere cortical atrophy (Figure 4B; $P = 0.037$). There were no correlations between the HCMV pp65 staining intensity and other clinical parameters.

## DISCUSSION

RE is now confirmed as an inflammatory autoimmune neurological syndrome that can be triggered by exogenous antigens and somehow mediated by Tlymphocyte cytotoxicity as well as autoantibodies (Li et al., 1997; Gahring et al., 2001; Bauer et al., 2002; Bien et al., 2002a; Schwab et al., 2009). However, virus infection, as an important potential initiating factor (Merkler et al., 2006; Chen et al., 2016), has not been carefully studied. In this study, we found significantly elevated pp65 expression in patients with RE in comparison with those in the control group. Additionally, patients with RE showing stronger pp65 staining exhibited a younger age of disease onset, more rapid clinical course, and more severe hemisphere atrophy compared with those having weakly positive or negative pp65 staining. In combination with the frequent history of HCMV infection before

Figure 2. Expression of HCMV pp65 protein in patients with RE. The pp65 protein was detected by IHC. Representative images are shown at low (200×, upper panels) or high (400×, lower panels) magnification. Positive staining of pp65 was predominantly observed in the cytoplasm of cells (arrows).

Table 2. Summary of IHC and ISH data for brain tissues of patients with and without RE

| | IHC | | ISH | |
|---|---|---|---|---|
| | RE (n = 26) | Non-RE (n = 20) | RE (n = 26) | Non-RE (n = 20) |
| Positive | 23 (88.5%) | 2 (10%) | 18 (69.2%) | 0 |
| Strong | 9 (34.6%) | 0 | 8 (30.8%) | 0 |
| Moderate | 9 (34.6%) | 0 | 6 (23.1%) | 0 |
| Weak | 5 (19.2%) | 2 (10%) | 4 (15.4%) | 0 |
| Negative | 3 (11.5%) | 18 (90%) | 8 (30.8%) | 20 (100%) |

Figure 3. Expression of HCMV pp65 DNA in patients with RE. HCMV pp65 DNA was detected by ISH. Representative images are shown at low (200×, upper panels) or high (400×, lower panels) magnification. Positive staining of pp65 DNA was predominantly observed in the cytoplasm and nucleus of cells (arrows).

Table 3. Comparison of IHC versus ISH results in this study

| Cases | IHC (positive) | IHC (negative) | Total |
|---|---|---|---|
| ISH (positive) | 18 | 0 | 18 |
| ISH (negative) | 5 | 3 | 8 |
| Total | 23 | 3 | 26 |

RE onset, these results suggested a correlation between the occurrence and development of RE and HCMV infection.

Previously, several groups have detected nucleic acids of EBV or HCMV in brain samples from patients with RE by ISH and/or PCR (Walter and Renella, 1989), implying the involvement of virus infections in RE development and progression. However, there has been little evidence of the presence of viral antigens in the brain tissues of patients with RE, and no reports have demonstrated thesignificant associations between the presence of viral components and clinical parameters. In this study, we detected, for the first time, not only a significantly elevation of HCMV pp65 antigen and DNA in patients with RE compared with that in controls, but also the association between HCMV pp65 expression level in the brain tissue and disease parameters inpatients with RE. High levels of HCMV pp65 antigen in patients with RE was closely related to a short duration of the prodromal stage, more severe hemisphere atrophy, and younger age at seizure onset. Therefore, our results, along with other reports, have further supported the hypothesis that there is a potential link between HCMV infections and disease progression of RE. However, additional studies are needed to test this hypothesis. A previous study reported no detection of HCMV, EBV, or HSV infections in seven RE cases using ISH (Atkins et al., 1995). According to reports and our experience, we believe that the reasons for these discrepancies may be related to differences in epidemiology, study populations, sample preparation, and detection techniques as well as the small sample sizes of the studies.

Figure 4. Association between HCMV pp65 antigen expression and the clinical parameters of patients with RE. Patients with RE were divided into group A (nega-tive or weakly positive for pp65 staining) and group B (strongly and moderately positive for pp65 staining). Associations of prodromal stage duration, age at seizure onset (A), and severe atrophy grade (B) with pp65 staining. *Atrophy grade (AG) was defined by the following principles: no (0 point), no enlargement of lateral ventricle and hemispheric sulcus; mild (1 point), widening of the hemispheric sulci without obvious enlargement of lateral ventricles; moderate (2 points), obvious widening of the hemispheric sulci with mild to moderate enlargement of lateral ventricles (less than double wide than contralateral in axial view); severe (3 points), obvious widening of the hemispheric sulci with severe enlargement of lateral ventricles (more than twice as wide as the contralateral axial view).

The mechanism by which HCMV is involved in the occurrence of RE is not well understood. HCMV and other viruses detected in RE brain tissues are highly prevalent in the general population (Britt, 2015; Looker et al., 2015). Since the average age of seizure onset in patients with RE was 5.8 years, we speculated that congenital and/or neonatal HCMV infections occurred in patients with RE. HCMV pp65 is an abundant structural protein of the virion and is often used as an indicator of the infection. Generally, during primary infection, viral antigens would be recognized by T cells, and a pool of memory T cells would be formed. The responses from memory T cells may damage neurons when being triggered by a secondary virus infection after birth and thus, could be linked to RE occurrence and progression. However, more mechanistic studies are required to further clarify how virus infection triggers the occurrence of RE.

In conclusion, elevated expression of antigen and nucleic acid of HCMV pp65 was detected in the brain tissues of patients with RE, and an abundance of HCMV antigen was found to be correlated with the duration of the prodromal stage, age of seizure onset, and hemisphere atrophy. These results suggested that HCMV may act as a trigger for RE and thereby participate in the occurrence and progression of RE. Additionally, antiviral therapy may be a potential strategy for the prevention and treatment of RE.

## ACKNOWLEDGMENTS

This work was supported by the following funds: the National Natural Science Foundation of China (81571275), the Beijing Municipal Natural Science Foundation (7144217), the Capital Applied Clinic Research Programs of Science and Technology (Z131107002213171), the Beijing Rising-star Plan of Science and Technology (Z141107001814042), the Open Research Fund of the Beijing Key Laboratory of Epilepsy Research (No. 2014DXBL02), Capital Medical University (15JL08), Scientific Research Common Program of Beijing Municipal Commission of Education (KM201610025001), Scientific Research Foundation for the Returned Overseas Chinese Scholars, State Education Ministry (2014 1685).

## COMPLIANCE WITH ETHICS GUIDELINES

The authors declare that they have no conflict of interest. This study was approved by the Ethics Committee of Sanbo Brain Hospital, Capital Medical University (2013 061801), and written informed consent was obtained from all participants or their guardians prior to the study.

## AUTHOR CONTRIBUTIONS

JA, GML, YSW conceived and designed the experiments. YZ, YSW, SCC performed the experiments. YSW, SCC analyzed the data. YGG, CQL, TFL contributed reagents/materials/analysis tools. YZ, YSW, SCC wrote the manuscript. YSW, SCC prepared the figures and/or tables. JA, GML checked and finalized the manuscript. All authors read and approved the final manuscript.

## OPEN ACCESS

This article is distributed under the terms of the Creative Commons Attribution 4.0 International License (http://creative commons.org/licenses/by/4.0/), which permits unrestricted use, distribution, and reproduction in any medium, provided you give appropriate credit to the original author(s) and the source, provide a link to the Creative Commons license, and indicate if changes were made.

## References

[CR1] Atkins MR, Terrell W, Hulette CM (1995). Rasmussen’s syndrome: a study of potential viral
etiology. Clin Neuropathol.

[CR2] Bauer J, Bien CG, Lassmann H (2002). Rasmussen’s encephalitis: a role for autoimmune
cytotoxic T lymphocytes. Curr Opin Neurol.

[CR3] Bien CG, Bauer J, Deckwerth TL, Wiendl H, Deckert M, Wiestler OD, Schramm J, Elger CE, Lassmann H (2002). Destruction of neurons by cytotoxic T cells: a new
pathogenic mechanism in Rasmussen’s encephalitis. Ann Neurol.

[CR4] Bien CG, Granata T, Antozzi C, Cross JH, Dulac O, Kurthen M, Lassmann H, Mantegazza R, Villemure JG, Spreafico R, Elger CE (2005). Pathogenesis, diagnosis and treatment of Rasmussen
encephalitis: a European consensus statement. Brain.

[CR5] Bien CG, Widman G, Urbach H, Sassen R, Kuczaty S, Wiestler OD, Schramm J, Elger CE (2002). The natural history of Rasmussen’s
encephalitis. Brain.

[CR6] Britt W (2015). Controversies in the natural history of congenital
human cytomegalovirus infection: the paradox of infection and disease in
offspring of women with immunity prior to pregnancy. Med Microbiol Immunol.

[CR7] Chen S, Chen S, Guan Y, Zhang Y, Qi X, An J, Wang Y, Luan G (2016). Elevated expression of human papillomavirus antigen
in brain tissue of patients with Rasmussen’s encephalitis. Epilepsy Res.

[CR8] Farrell MA, Cheng L, Cornford ME, Grody WW, Vinters HV (1991). Cytomegalovirus and Rasmussen’s
encephalitis. Lancet.

[CR9] Gahring L, Carlson NG, Meyer EL, Rogers SW (2001). Granzyme B proteolysis of a neuronal glutamate
receptor generates an autoantigen and is modulated by
glycosylation. J Immunol.

[CR10] Guan Y, Zhou J, Luan G, Liu X (2014). Surgical treatment of patients with Rasmussen
encephalitis. Stereotact Funct Neurosurg.

[CR11] Jay V, Becker LE, Otsubo H, Cortez M, Hwang P, Hoffman HJ, Zielenska M (1995). Chronic encephalitis and epilepsy (Rasmussen’s
encephalitis): detection of cytomegalovirus and herpes simplex virus 1 by
the polymerase chain reaction and in situ hybridization. Neurology.

[CR12] Li Y, Uccelli A, Laxer KD, Jeong MC, Vinters HV, Tourtellotte WW, Hauser SL, Oksenberg JR (1997). Local-clonal expansion of infiltrating T lymphocytes
in chronic encephalitis of Rasmussen. J Immunol.

[CR13] Looker KJ, Magaret AS, Turner KM, Vickerman P, Gottlieb SL, Newman LM (2015). Global estimates of prevalent and incident herpes
simplex virus type 2 infections in 2012. PLoS One.

[CR14] Luan G, Gao Q, Guan Y, Zhai F, Zhou J, Liu C, Chen Y, Yao K, Qi X, Li T (2013). Upregulation of adenosine kinase in Rasmussen
encephalitis. J Neuropathol Exp Neurol.

[CR15] Luan G, Gao Q, Zhai F, Chen Y, Li T (2016). Upregulation of HMGB1, toll-like receptor and RAGE
in human Rasmussen’s encephalitis. Epilepsy Res.

[CR16] Manicklal S, Emery VC, Lazzarotto T, Boppana SB, Gupta RK (2013). The "silent" global burden of congenital
cytomegalovirus. Clin Microbiol Rev.

[CR17] Merkler D, Horvath E, Bruck W, Zinkernagel R D l, Torre JC, Pinschewer DD (2006). "Viral deja vu" elicits organ-specific immune
disease independent of reactivity to self. J Clin Invest.

[CR18] Pardo CA, Vining EP, Guo L, Skolasky RL, Carson BS, Freeman JM (2004). The pathology of Rasmussen syndrome: stages of
cortical involvement and neuropathological studies in 45
hemispherectomies. Epilepsia.

[CR19] Power C, Poland SD, Blume WT, Girvin JP, Rice GP (1990). Cytomegalovirus and Rasmussen’s
encephalitis. Lancet.

[CR20] Rasmussen T, Olszewski J, Lloydsmith D (1958). Focal seizures due to chronic localized
encephalitis. Neurology.

[CR21] Rogers SW, Andrews PI, Gahring LC, Whisenand T, Cauley K, Crain B, Hughes TE, Heinemann SF, McNamara JO (1994). Autoantibodies to glutamate receptor GluR3 in
Rasmussen’s encephalitis. Science.

[CR22] Schwab N, Bien CG, Waschbisch A, Becker A, Vince GH, Dornmair K, Wiendl H (2009). CD8+ T-cell clones dominate brain infiltrates in
Rasmussen encephalitis and persist in the periphery. Brain.

[CR23] Takahashi Y, Matsuda K, Kubota Y, Shimomura J, Yamasaki E, Kudo T, Fukushima K, Osaka H, Akasaka N, Imamura A, Yamada S, Kondo N, Fujiwara T (2006). Vaccination and infection as causative factors in
Japanese patients with Rasmussen syndrome: molecular mimicry and HLA class
I. Clin Dev Immunol.

[CR24] Tandon R, Mocarski ES (2012). Viral and host control of cytomegalovirus
maturation. Trends Microbiol.

[CR25] Varadkar S, Bien CG, Kruse CA, Jensen FE, Bauer J, Pardo CA, Vincent A, Mathern GW, Cross JH (2014). Rasmussen’s encephalitis: clinical features,
pathobiology, and treatment advances. Lancet Neurol.

[CR26] Walter GF, Renella RR (1989). Epstein-Barr virus in brain and Rasmussen’s
encephalitis. Lancet.

